# Values and diagnostic accuracy of sensory nerve action potentials in control participants and participants with diabetes with and without clinical diabetic neuropathy, based on neuropathy scale measurements

**DOI:** 10.1002/brb3.3423

**Published:** 2024-02-13

**Authors:** Ahmad R. Abuzinadah, Moafaq S. Alrawaili, Aysha A. Alshareef, Hussien S. Alkully, Haneen Milyani, Bashayr Alamri, Weam Alshora, Ahmed K. Bamaga

**Affiliations:** ^1^ Department of Neurology, Faculty of Medicine King Abdulaziz University Jeddah Saudi Arabia; ^2^ Neuromuscular Medicine Unit King Abdulaziz University Hospital, King Abdulaziz University Jeddah Saudi Arabia; ^3^ Neurology Section, Department of Neurosciences King Faisal Specialist Hospital and Research Centre Jeddah Saudi Arabia; ^4^ Neurophysiology Department, National Neuroscience Institute King Fahad Medical City Riyadh Saudi Arabia; ^5^ Internal Medicine Department, Neurology division King Fahad General Hospital Jeddah Saudi Arabia; ^6^ Department of Family Medicine King Abdulaziz University Hospital, King Abdulaziz University Jeddah Saudi Arabia; ^7^ Pediatric Neurology Unit, Department of Pediatric, Faculty of Medicine King Abdulaziz University Jeddah Saudi Arabia; ^8^ Division of Pediatric Neurology, Department of Pediatrics King Faisal Specialist Hospital and Research Centre Jeddah Saudi Arabia

**Keywords:** diabetes, diagnosis, neuropathy, screening, sensitivity

## Abstract

**Background:**

The assessment of the normative values of sensory nerve action potentials (SNAP) and their diagnostic accuracies using validated neuropathy‐assessment tools to classify participants into groups with and without neuropathy was not previously described in the literature.

**Methods:**

The Utah Early Neuropathy Scale (UENS), Michigan neuropathy‐screening instrument, and nerve conduction data were collected prospectively. We described and compared the values of the sural, superficial peroneal sensory (SPS), and superficial radial SNAP amplitude in different age groups for three groups. Group 1 (G1)—control participants (UENS <5), group 2 (G2)—participants with diabetes without clinical diabetic neuropathy (UENS <5), and group 3 (G3)—participants with clinical diabetic neuropathy (UENS ≥5). We also described the diagnostic accuracy of single‐nerve amplitude and a combined sensory polyneuropathy index (CSPNI) that consists of four total points (one point for each of the following nerves if their amplitude was <25% lower limit of normal: right sural, left sural, right SPS, and left SPS potentials).

**Results:**

We assessed 135 participants, including 41, 37, and 57 participants in G1, G2, and G3, respectively, with age median (interquartile ranges) of 51 (45–56), 47 (38–56), and 54 (51–61) years, respectively, whereas 19 (46.3%), 18 (48.7%), and 32 (56.14%) of them were males, respectively. The sensitivity, specificity, positive predictive value (PPV), and negative predictive value (NPV) scores were 68.4%, 92.3%, 86.7%, and 80% for the sural amplitude; 86%, 58.3%, 62%, and 84% for the SPS amplitude; 66.7%, 94.4%, 90.5%, and 78.2% for the CSPNI of 3; and 54.4%, 98.6%, 96.9%, and 73.2% for the CSPNI of 4, respectively.

**Conclusion:**

Sural nerve had a high specificity for neuropathy; however, the CSPNI had the highest specificity and PPV, whereas the SPS had the highest sensitivity and NPV.

## Abstract 2

We described the sensitivity, specificity, positive predictive value, and negative predictive values of sensory nerve action potentials. We used a validated neuropathy scales to classify our participants into neuropathy and no neuropathy groups. These diagnostic accuracies were not reported in the previous literature.

## 1 INTRODUCTION

Peripheral polyneuropathy affects a large proportion of the population, with a prevalence of 2%–8%, and diabetes mellitus (DM) is the most common cause of peripheral neuropathy (Beghi & Monticelli, 1995; Smith & Singleton, 2012). Peripheral neuropathy is reported in 50% of patients diagnosed with DM (Tesfaye et al., 2010). Early diagnosis may delay progression and reduce the complications of neuropathy (Rubio et al., 2014). Hence, several guidelines have recommended annual screening for neuropathy among patients with diabetes (American Diabetes Association, 2012; Boulton et al., 2005; Pérez‐Panero et al., 2019). In particular, DM is associated with neuropathies, and lifestyle interventions may change the course of the disease (Smith et al., 2006). Neuropathy diagnoses are confirmed using nerve conduction studies (NCSs) (Dyck et al., 2003). Length‐dependent neuropathy initially affects the sensory nerves of the feet because of dying‐back phenomena (Uluc et al., 2008). Hence, the sural and superficial peroneal sensory (SPS) nerves are used to diagnose peripheral neuropathy. As sural nerves are less frequently affected by compression, they may be more accurate for diagnosing polyneuropathies (Burke et al., 1974; Uluc et al., 2008).

Three aspects could be added to previous studies regarding the sensitivity of NCSs. First, most previous studies did not use validated clinical neuropathy scales to differentiate patients with neuropathy from those without neuropathy. Scales that could be used to screen for neuropathy include the Michigan neuropathy‐screening instrument (MNSI) (Feldman et al., 1994) and the Utah Early Neuropathy Scale (UENS) (Singleton et al., 2008). Compared with other neuropathy scales, the UENS is more accurate for diagnosing neuropathy (Singleton et al., 2008). Kural et al. (2017) investigated the utility of distal sensory nerves in diagnosing peripheral neuropathy and measured UENS in their participants; however, they did not use UENS in defining neuropathy. Although UENS was not validated to be used in diagnosing cryptogenic sensory polyneuropathy, however, it has been demonstrated that UENS can be used to differentiate between healthy control participants and cases of neuropathy related to diabetes or glucose intolerance (Kural et al., 2017; Zilliox et al., 2015). Second, the positive predictive value (PPV) and the negative predictive value (NPV) have been minimally described in the literature. Predictive values are statistical measures that indicate how accurately a test predicts the presence or absence of a disease. The PPV measures how likely a positive test in a particular patient indicates that the patient has the disease, whereas the NPV measures how likely a negative test in a particular patient indicates that patient does not have the disease (Preston & Shapiro, 2013). Third, the composite score is more accurate than a single‐nerve conduction parameter (Dyck et al., 2003; Heise et al., 2012). Although several composite scores have been described, they comprise many parameters with variable cutoff values that affect their use in clinical practice (Dyck et al., 2003; Heise et al., 2012).

Our aim was to determine the sensory nerve action potential (SNAP) ranges of the sural, SPS, and superficial radial sensory nerves among three groups of participants, namely, group 1 (G1)—control participants, group 2 (G2)—participants with diabetes without clinical diabetic neuropathy, and group 3 (G3)—participants with clinical diabetic neuropathy, using validated neuropathy scales to classify participants. We also aimed to describe the sensitivity, specificity, PPV, and NPV of single sensory nerves and the combined NCS parameters stratified by age groups (AGs).

## 2 METHODS

### 2.1 Study design and participants

Data were collected from prospective cross‐sectional studies conducted between June 2018 and February 2021 at King Abdulaziz University Hospital (KAUH). The KAUH institutional review board approved the protocol (reference 389‐23). Each participant provided informed consent. Our inclusion criteria were as follows: (1) age range between 18 and 75 years and (2) no history of any other neurological diagnosis associated with sensory or motor symptoms, such as multiple sclerosis or stroke.

### 2.2 Data collection

To standardize the data collection procedure, neurologists and senior neurology residents (physicians who had completed 2 years of the neurology residency program) were trained to use neuropathy scales. Participants who met our inclusion criteria and who were referred to nerve conduction study testing for a suspicion of neuropathy were recruited. The following neuropathy scales were collected: the MNSI (questionnaire [MNSIq‐Ar] and examination [MNSIe]) and the UENS (Feldman et al., 1994; Meijer et al., 2002; Singleton et al., 2008). An electrophysiology technician who was blinded to the diagnosis performed NCSs bilaterally for the sural, SPS, and superficial radial sensory nerves.

### 2.3 Variables and group definitions

Patients with DM included participants diagnosed with DM by a specialized physician and either an elevated hemoglobin A1c level (≥6.5%) or the use of hypoglycemic agents.

Participants were classified into three groups. G1 included control participants defined as participants without DM with a UENS score of <5. G2 included participants with diabetes without clinical diabetic neuropathy (UENS score of <5). G3 included participants with clinical diabetic neuropathy (UENS score of ≥5). Six participants were diagnosed with DM with a UENS <5; however, their MNSI score was >7.5, which meant they had a > 90% probability of having diabetes neuropathy (Abuzinadah et al., 2021). These six participants were excluded from the primary analysis and included in G2 in the sensitivity analysis (Supplementary Materials: Appendix A).

### 2.4 Sensory nerve stimulation and recording protocol

We used the standard parameters for sensory nerve conduction testing utilizing electrical pulses of 0.1 ms duration, generated at a frequency of 1 Hz, and with current ranging from 5 to 60 mA to achieve supramaximal stimulation, defined as the point before muscle twitching occurs. The current was gradually increased in increments of 3–5 mA from 0 mA until the recorded sensory potential peaked. To enhance the signal‐to‐noise ratio and obtain a robust measurement, we used the average of responses from at least 3 to 5 supramaximal stimulations. Latency measurements were performed using established protocols. For biphasic SNAPs, the peak latency was measured at the midpoint of the first negative peak. Amplitude was consistently measured from the baseline to the first negative peak (Buschbacher & Prahlow, 2006; Daube & Rubin, 2009; Preston & Shapiro, 2013). The Natus Dantec machine (Middleton, WI 53562) and Natus Dantec keypoint software were used for data acquisition and analysis. We used disposable adhesive surface electrodes (101169; Natus) for all measurements. All NCS measurements were performed at a limb temperature of 33–34°C. Skin temperature of the limbs was measured before conducting the NCS; if the limb temperature was <33°C, it was warmed to the required temperature.

For the sural nerve amplitude (SNA), we stimulated at the posterior–lateral calf, 14 cm from the recording site, and recorded the response posterior to the lateral malleolus. For the SPS nerve amplitude (SPSA), we stimulated at the lateral calf, 14 cm from the recording site, and recorded the response between the tibialis anterior tendon and the lateral malleolus. For the superficial radial sensory nerve amplitude (SRSA), we stimulated over the distal‐mid radius, 10 cm from the recording site, and recorded over the extensor pollicis longus tendon to the thumb. The sural to radial amplitude ratio (SRAR) was achieved by dividing the SNA by the SRSA.

### 2.5 Objectives

The primary objective of this study was to establish the SNAP ranges of SNA, SPSA, and SRSA among the three groups defined above. The range was based on average measurements between the right and left sides of the body. The secondary objective was to establish sensory potential ranges of the three groups defined above for each (AG) as follows: age (AG) 1: <40 years, (AG) 2: 40–49 years, (AG) 3: 50–59 years, and (AG) 4: 60–75 years.

We reported the diagnostic accuracy of using single nerve (either SNA, SPSA, or SRAR) to diagnose peripheral neuropathy using the receiver operator characteristic (ROC) area under the curve, as well as the sensitivity, specificity, PPV, and NPV. The cutoff value was chosen based on the ROC. We proposed a new simplified composite score, the combined polyneuropathy sensory index (CPNSI), and evaluated its diagnostic accuracy. CPNSI consisted of four total points (one point for each of the following nerve potentials in which their amplitude values were <25% lower limit of normal [LLN]: right sural, left sural, right SPS, and left SPS potentials). In CPNSI, each sural nerve was assigned one point if the value was less than 8.5 μV, and each superficial peroneal potential was assigned one point if the value was less than 4.5 μV, with a maximum of four points. These cutoff values used in CPNSI were chosen because they represent the 25% LLN.

### 2.6 Sensitivity analysis

We reported the diagnostic accuracy after excluding the control participants G1. We also re‐performed all the analysis, including the six participants with a UENS <5 and MNSI of >7.5. Moreover, they were included in G2.

### 2.7 Statistical analysis

The demographic features were described using medians, interquartile ranges (IQR), and frequencies. We used the Mann–Whitney *U* tests, Fisher's exact test, and *χ* ^2^ to compare the medians and proportions as appropriate. The diagnostic accuracy of diabetic neuropathy was evaluated using the ROC area. The diagnostic properties included sensitivity, specificity, PPV, and NPV. Statistical analyses were performed using STATA version 13 (Stata Corp).

## 3 RESULTS

Between June 2018 and February 2021, 135 patients (69 of whom were men) participated in the study, of these 78 without neuropathy (41 without diabetes G1 and 37 with diabetes G2), and 57 participants with clinical diabetic neuropathy G3. The participants’ characteristics are summarized in Table 1. The gender distribution was equal among the three groups. The median age of participants in the G3 group was higher (54 years) than that of G1 participants (51 years) and the G2 participants (47 years), (*p* < .05). The median duration of diabetes was 5 years in the G2 group, which is less than half the duration for the G3 group participants (15 years). The 50–59 years AG represented the highest proportion of participants in each group (*p* < .05). Those aged 60–75 years were more prevalent in G3 (29.8%) than in G1 (7.3%) and G2 (2.7%) (*p* < .05). To adjust for this imbalance, an analysis was performed for each AG.

**TABLE 1:** Participants’ characteristics.

	No neuropathy (G1) (control participants) *N* = 41	No neuropathy (G2) (participants with diabetes) *N* = 37	Diabetic neuropathy (G3) *N* = 57	G1 versus G3^a^	G2 versus G3^a^	All groups^b^
Age, median (IQR)	51 (45–56)	47 (38–56)	54 (51–61)	0.0103	0.001	0.0014
Male, *n* (%)	19 (46.3)	18 (48.7)	32 (56.14)	0.226	0.309	0.622
Diabetes, *n* (%)	0	37 (100)	57 (100)	–	–	0.00
Diabetes duration (years), median (IQR)	0	5.5 (2–12.5)	15 (10–21.5)	–	0.0001	0.0001
UENS, median (IQR)	0 (0–0)	0 (0–2)	10 (6–16)	0.000	0.000	0.0001
MNSI, median (IQR)	0 (0–1.5)	2 (1–5)	10.5 (8–13)	0.000	0.000	0.0001
Burning feet, n (%)	3 (7.3)	12 (32.4)	38 (66.7)	0.000	0.001	0.000
AG1: 60–75 (years) *n* (%)	3 (7.3)	1 (2.7)	17 (29.8)	0.003	0.000	0.00
AG2: 50–59 (years) *n* (%)	20 (48.8)	15 (40.6)	28 (49.12)			
AG3: 40–49 (years) *n* (%)	18 (43.9)	11 (29.7)	10 (17.5)			
AG4: <40 (years) *n* (%)	0	10 (27.0)	2 (3.5)			

*Note*: G1 versus G2 comparison *p* value were >.05 except MNSI comparison, burning feet and age group *p* values were .00, .005, and .001, respectively.

Abbreviation: AG, age group; MNSI, Michigan neuropathy‐screening instrument; UENS, Utah Early Neuropathy Scale.

^a^ Mann–Whitney *U* test, Fisher's exact test for proportion comparisons.

^b^ Kruskal–Wallis rank test for median comparisons, Fisher's exact test for proportion comparisons.

### 3.1 Range of sensory potential parameters in the neuropathy and no neuropathy groups

There was a significant difference in all SNAP on comparing the G3 group participants with the G2 or G1 group participants, as shown in Table 2. On the other hand, there was no difference between the G1 group and the G2 group participants. The 25% lower limits (LLs) of IQR for the sural nerve potentials were greater than 7.5 μV in all AGs within G1 and greater than 6.6 μV in G2. However, within the neuropathy group, the 75% upper limit of IQR was less than 10 μV in all the AGs except for (AG) 1 (<40 years) in which it was less than 16.5 μV. The 25% LL of IQR for the SPSA was greater than 5.5 μV in G1 and greater than 3.5 μV in G2 all AGs except for G1 AG4 (60–75 years), in which it was greater than 0.8 μV. The 25% lower limit of IQR for the superficial radial sensory nerve potentials was greater than 24.5 μV in all AGs in G1 and G2, except for AG 3 in G2 (>18 μV).

**TABLE 2:** Range of the amplitude of the sensory potentials in control participants and participants with diabetes with and without clinical diabetic neuropathy.

Age group (AG)	No neuropathy (G1) (healthy controls), μV median (IQR)/5% LLN	No neuropathy (G2) (participants with DM), μV median (IQR)/5% LLN	Diabetic neuropathy (G3), μV median (IQR)	*p* Value^a^ G1 versus G3	*p* Value^a^ G2 versus G3	*p* Value^b^ (all groups)
	Sural nerve potential (μV)					
Whole cohort	11.9 (9.15–15.25)/7.4	10.5 (8.55–15.75)/4.6	4.05 (0–8.95)	.000	.000	.000
AG1 <40 years	–	15.25 (10.1–21.8)/5.95	8.18 (0–16.35)	–	.39	.39
AG2 40–49 years	13.38 (9.95–17.35)/7.55	11 (8.5–15.4)/4.65	2.03 (0–7.9)	.000	.013	.0036
AG3 50–59 years	12.2 (8.68–14.63)/6	8.85 (8.5–14.2)/3.95	4.37 (0–9.57)	.000	.004	.0002
AG4 60–75 years	7.55 (7.5–11.6)/7.5	6.65 (6.65–6.65)/6.65	0 (0–8.75)	.2	.61	.379
	Superficial peroneal sensory nerve potential (μV)					
Whole cohort	7.95 (6.25–10)/3.65	5.5 (4.25–10.5)/2.14	0 (0–4.3)	.000	.000	.0001
AG1 <40 years	–	8.7 (4.65–10.6)/2.5	1.87 (0–3.75)	–	.086	.086
AG2 40–49 years	8.4 (6.65–10)/3.65	6.65 (4.6–9.4)/2.2	3.18 (0–4)	.000	.013	.0008
AG3 50–59 years	7.7 (5.7–9.95)/3.85	5.05 (3.55–13.5)/1.4	1.55 (0–4.33)	.000	.001	.0001
AG4 60–75 years	5.07 (0.83–9.3)/0.83	5.15 (5.15–5.15)/5.15	0 (0–5.3)	.12	.42	.26
	Superficial radial sensory nerve potential (μV)					
Whole cohort	29.8 (26.3–37.55)/20.7	28 (24–39.4)/15.8	18.1(10.85–26.55)	.000	.000	.000
AG1<40 years	–	32.3 (26.9–42.75)/21.75	16.35 (6.5–26.2)		.099	.099
AG2 40–49 years	30.4 (26.3–40.45)/18.75	27.55 (25.45–32.05)/20	19.27 (10.2–33.15)	.021	.11	.056
AG3 50–59 years	28.6 (25.95–32.05)/21.65	25.7 (18.05–40.9)/14.7	18.7 (11.3–27.6)	.002	.013	.0.003
AG4 60–75 years	37.5 (37.5–37.5)/37.55	24.9 (24.9–24.9)/24.9	13.45 (10.85–20.85)	.107	.26	.15
	Sural to radial ratio					
Whole cohort	0.39 (0.33–0.51)/0.23	0.36 (0.3–0.47)/0.23	0.21 (0–0.41)	.000	.000	.000
AG1 <40 years	–	0.36 (0.32–0.74)/0.27	0.31 (0–0.62)	–	.47	.47
AG2 40–49 years	0.43 (0.35–0.55)/0.23	0.35 (0.28–0.5)/0.23	0.11 (0–0.32)	.001	.013	.002
AG3 50–59 years	0.41 (0.29–0.51)/0.27	0.41 (0.31–0.46)/0.21	0.22 (0–0.46)	.021	.053	.03
AG4 60–75 years	0.2 (0.2–0.2)/0.2	0.27 (0.27–0.27)/0.27	0.15 (0–0.41)	1.00	1.0	.98

*Note*: All comparisons between G1 and G2 were statistically insignificant at >.05.

Abbreviations: AG, age group; IQR, interquartile range; LLN, lower limit of normal.

^a^ Mann–Whitney *U* test.

^b^ Kruskal–Wallis test.

### 3.2 Diagnostic accuracy of peripheral neuropathy

Using a single SNA measurement lower than 5.1 μV produced an excellent specificity (>90%) in the entire cohort and in each AG, as shown in Table 3. The sensitivity for the whole cohort and for each AG was 64%–80%, except for AG1, where it was 50%. The PPV was moderate to high (80%–100%), except in AG1, where it was low (50%). The NPV was high (≥90%) in the younger AGs (<50 years) and low (40%–78%) in the older AGs (50–75 years). The single SPSA measurement (<4.5 μV) produced a high sensitivity (100%) and a high NPV (100%) in participants younger than 40 years, whereas in the older AGs (40–75 years), the sensitivity was 80%–90%, and the NPV was 40%–95%. The specificity and PPV of single SPSA were low, as shown in Table 3.

**TABLE 3:** Diagnostic accuracy of the amplitude of the sensory nerve action potentials (no neuropathy group included control participants and participants with diabetes without neuropathy).

Age group	ROC	Sensitivity	Specificity	PPV	NPV
	Single sural nerve potential <5.1 μV				
Whole cohort	0.804 (0.736–0.871)	68.4% (54.8%–80.1%)	92.3% (84%–97.1%)	86.7% (73.2%–94.9%)	80% (70.2%–87.7%)
Age group 1 <40 years	0.7 (0.2–1)	50% (1.26%–98.7%)	90% (55.5%–99.7%)	50% (1.26%–98.7%)	90% (55.5%–99.7%)
Age group 2 40–49 years	0.866 (0.727–1)	80% (44.4%–97.5%)	93.1% (77.2%–99.2%)	80% (44.4%–97.5%)	93.1% (77.2%–99.2%)
Age group 3 50–59 years	0.796 (0.697–0.896)	67.9% (47.6%–84.1%)	91.4% (76.9%–98.2%)	86.4% (65.1%–97.1%)	78% (62.4%–89.4%)
Age group 4 60–75 years	0.824 (0.706–0.941)	64.7% (38.3%–85.8%)	100% (39.8%100%)	100% (71.5%–100%)	40% (12.2%–73.8%)
	Single superficial peroneal sensory <4.5 μV				
Whole cohort	0.721 (0.648–0.795)	86% (74.2%–93.7%)	58.3% (46.1%–69.8)	62% (50.4%–72.7%)	84% (70.9%–92.8%)
Age group 1 <40 years	0.8 (0.64–0.96)	100% (15.8%0–100%)	60% (26.2%–87.8%)	33.3% (4.33%–77.7%)	100% (54.1%–100%)
Age group 2 40–49 years	0.778 (0.646–0.909)	90% (55.5%–99.7%)	65.5% (45.7%–82.1%)	47.4% (24.4%–71.1%)	95% (75.1%–99.9%)
Age group 3 50–59 years	0.679 (0.566–0.791)	85.7% (67.3%–96%)	50% (31.3%–68.7%)	61.5% (44.6%–76.6%)	78.9% (54.4%–93.9%)
Age group 4 60–75 years	0.745 (0.405–1)	82.4% (56.6%–96.2%)	66.7% (9.43%–99.2%)	93.3% (68.1%–99.8%)	40% (5.27%–85.3%)
	Sural‐to‐radial ratio <0.2				
Whole cohort	0.74 (0.67–0.81)	48% (33.7%–62.6%)	100% (94.8%–100%)	100% (85.8%–100%)	72.6% (62.5%–81.3%)
Age group 1 <40 years	0.75 (0.26–1)	50% (1.26%–98.7%)	100% (66.4%–100%)	100% (2.5%–100%)	90% (55.5%–99.7%)
Age group 2 40–49 years	0.75 (0.587–0.913)	50% (18.7%–81.3%)	100% (88.1%–100%)	100% (47.8%–100%)	85.3% (68.9%–95%)
Age group 3 50–59 years	0.731 (0.633–0.828)	46.2% (26.6%–66.6%)	100% (88.1%–100%)	100% (73.5%–100%)	67.4% (51.5%–80.9%)
Age group 4 60–75 years	0.75 (0.602–0.898)	50% (21.1%–78.9%)	100% (15.8%–100%)	100% (54.1%–100%)	25% (3.19%–65.1%)
	Combined polyneuropathy sensory index (CPNSI) (right and left sural <8.5, right and left superficial peroneal <4.5)				
1 out of 4 sensory nerves	0.664 (0.596–0.733)	91.2% (80.7%–97.1%)	41.7% (30.2%–53.9%)	55.3% (44.7%–65.6%)	85.7% (69.7%–95.2%)
2 out of 4 nerves	0.765 (0.691–0.838)	80.7% (68.1%–90%)	72.2% (60.4%–82.1%)	69.7% (57.1%–80.4%)	82.5% (70.9%–90.9%)
3 out of 4 sensory nerves	0.806 (0.738–0.873)	66.7% (52.9%–78.6%)	94.4% (86.4%–98.5%)	90.5% (77.4%–97.3%)	78.2% (68%–86.3%)
4 out of 4 nerves	0.765 (0.698–0.832)	54.4% (40.7%–67.6%)	98.6% (92.5%–100%)	96.9% (83.8%–99.9%)	73.2% (63.2%–81.7%)
	Combined polyneuropathy sensory index (CPNSI) (3 out of 4; right and left sural <8.5, right and left superficial peroneal <4.5)				
Whole cohort	0.806 (0.738–0.873)	66.7% (52.9%–78.6%)	94.4% (86.4%–98.5%)	90.5% (77.4%–97.3%)	78.2% (68%–86.3%)
Age group 1 <40 years	0.75 (0.26–1)	50% (1.26%–98.7%)	100% (69.2%–100%)	100% (2.5%–100%)	90.9% (58.7%–99.8%)
Age group 2 40–49 years	0.833 (0.679–0.986)	70% (34.8%–93.3%)	96.6% (82.2%–99.9%)	87.5% (47.3%–99.7%)	90.3% (74.2%–98%)
Age group 3 50–59 years	0.789 (0.686–0.893)	67.9% (47.6%–84.1%)	90% (73.5%–97.9%)	86.4% (65.1%–97.1%)	75% (57.8%–87.9%)
Age group 4 60–75 years	0.824 (0.706–0.941)	64.7% (38.3%–85.8%)	100% (29.2%–100%)	100% (71.5%–100%)	33.3% (7.49%–70.1%)
	Combined polyneuropathy sensory index (CPNSI) (4 out of 4; right and left sural <8.5, right and left superficial peroneal <4.5)				
Whole cohort	0.765 (0.698–0.832)	54.4% (40.7%–67.6%)	98.6% (92.5%–100%)	96.9% (83.8%–99.9%)	73.2% (63.2%–81.7%)
Age group 1 <40 years	0.75 (0.26–1)	50% (1.26%–98.7%)	100% (69.2%–100%)	100% (2.5%–100%)	90.9% (58.7%–99.8%)
Age group2 40–49 years	0.75 (0.587–0.913)	50% (18.7%–81.3%)	100% (88.1%–100%)	100% (47.8%–100%)	85.3% (68.9%–95%)
Age group 3 50–59 years	0.733 (0.634–0.833)	50% (30.6%–69.4%)	96.7% (82.8%–99.9%)	93.3% (68.1%–99.8%)	67.4% (51.5%–80.9%)
Age group 4 60–75 years	0.824 (0.706–0.941)	64.7% (38.3%–85.8%)	100% (29.2%–100%)	100% (71.5%–100%)	33.3% (7.49%–70.1%)

Abbreviation: CPNSI, combined polyneuropathy sensory index; NPV, negative predictive value; PPV, positive predictive value; ROC, receiver operator characteristic.

### 3.3 Combined sensory polyneuropathy index (CSPNI)

The Combined sensory polyneuropathy index is a total of four points score with one point, which is given for each of the following nerve potentials in which their values were less than 25% LLN: right sural, left sural, right SPS, and left SPS potentials. The sensitivity and PPV increased as score increased. When one or two points out of four points were used, the specificity and PPV were less than 75% each, whereas both were higher than 90% when three points out of the four were used. Based on AGs, using three points produced over 87.5% PPV and about 90% NPV in the under‐50 AGs, whereas using four points produced over 90% PPV in the 50–75 AG; however, the NPV remained <80%.

### 3.4 Sensitivity analysis

The diagnostic accuracy of different SNAPs after excluding the control participants was similar to the analysis performed, including this group (Table 4). Similarly, when the six participants with UENS <5 and MNSI >7.5 were included in the G2 group, the SNAP ranges and diagnostic accuracy remained stable (Supplementary Materials: Appendix A).

**TABLE 4:** Diagnostic accuracy of the amplitude of the sensory nerve action potentials (no neuropathy group included only participants with diabetes without neuropathy and excluded control participants).

Age AG	ROC	Sensitivity	Specificity	PPV	NPV
	Single sural nerve potential <5.1 μV				
Whole cohort	0.775 (0.692–0.857)	68.4% (54.8%–80.1%)	86.5% (71.2%–95.5%)	88.6% (75.4%–96.2%)	64% (49.2%–77.1%)
AG1 <40 years	0.7 (0.2–1)	50% (1.26%–98.7%)	90% (55.5%–99.7%)	50% (1.26%–98.7%)	90% (55.5%–99.7%)
AG2 40–49 years	0.809 (0.632–0.986)	80% (44.4%–97.5%)	81.8% (48.2%–97.7%)	80% (44.4%–97.5%)	81.8% (48.2%–97.7%)
AG3 50–59 years	0.773 (0.647–0.898)	67.9% (47.6%–84.1%)	86.7% (59.5%–98.3%)	90.5% (69.6%–98.8%)	59.1% (36.4%–79.3%)
AG4 60–75 years	0.824 (0–1)	64.7% (38.3%–85.8%)	100% (2.5%–100%)	100% (71.5%–100%)	14.3% (0.361%–57.9%)
	Single superficial sensory peroneal <4.5 μV				
Whole cohort	0.673 (0.58–0.767)	86% (74.2%–93.7%)	48.6% (31.9%–65.6%)	72.1% (59.9%–82.3%)	69.2% (48.2%–85.7%)
AG1 <40 years	0.8 (0.64–0.96)	100% (15.8%–100%)	60% (26.2%–87.8%)	33.3% (4.33%–77.7%)	100% (54.1%–100%)
AG2 40–49 years	0.677 (0.494–0.86)	90% (55.5%–99.7%)	45.5% (16.7%–76.6%)	60% (32.3%–83.7%)	83.3% (35.9%–99.6%)
AG3 50–59 years	0.629 (0.484–0.773)	85.7% (67.3%–96%)	40% (16.3%–67.7%)	72.7% (54.5%–86.7%)	60% (26.2%–87.8%)
AG4 60–75 years	0.912 (0–1)	82.4% (56.6%–96.2%)	100% (2.5%–100%)	100% (76.8%–100%)	25% (0.631%–80.6%)
	Sural to radial ratio <0.2				
Whole cohort	0.74 (0.67–0.81)	48% (33.7%–62.6%)	100% (90.3%–100%)	100% (85.8%–100%)	58.1% (44.8%–70.5%)
AG1 <40 years	0.75 (0.26–1)	50% (1.26%–98.7%)	100% (66.4%–100%)	100% (2.5%–100%)	90% (55.5%–99.7%)
AG2 40–49 years	0.75 (0.587–0 .913)	50% (18.7%–81.3%)	100% (71.5%–100%)	100% (47.8%–100%)	68.8% (41.3%–89%)
AG3 50–59 years	0.731 (0.633–0.828)	46.2% (26.6%–66.6%)	100% (78.2%–100%)	100% (73.5%–100%)	51.7% (32.5%–70.6%)
AG4 60–75 years	0.75 (0.0–1)	50% (21.1%–78.9%)	100% (2.5%–100%)	100% (54.1%–100%)	14.3% (0.361%–57.9%)
	Combined polyneuropathy sensory index (CPNSI) (right and left sural <8.5, right and left superficial peroneal <4.5)				
1 out of 4 sensory nerves	0.632 (0.545–0.718)	91.2% (80.7%–97.1%)	35.1% (20.2%–52.5%)	68.4% (56.7%–78.6%)	72.2% (46.5%–90.3%)
2 out of 4 nerves	0.728 (0.634–0.821)	80.7% (68.1%–90%)	64.9% (47.5%–79.8%)	78% (65.3%–87.7%)	68.6% (50.7%–83.1%)
3 out of 4 sensory nerves	0.779 (0.699–0.859)	66.7% (52.9%–78.6%)	89.2% (74.6%–97%)	90.5% (77.4%–97.3%)	63.5% (49%–76.4%)
4 out of 4 nerves	0.758 (0.688–0.829)	54.4% (40.7%–67.6%)	97.3% (85.8%–99.9%)	96.9% (83.8%–99.9%)	58.1% (44.8%–70.5%)
	Combined polyneuropathy sensory index (CPNSI) (3 out of 4; right and left sural <8.5, right and left superficial peroneal <4.5)				
Whole cohort	0.779 (0.699–0.859)	66.7% (52.9%–78.6%)	89.2% (74.6%–97%)	90.5% (77.4%–97.3%)	63.5% (49%–76.4%)
AG1 <40 years	0.75 (0.26–1)	50% (1.26%–98.7%)	100% (69.2%–100%)	100% (2.5%–100%)	90.9% (58.7%–99.8%)
AG2 40–49 years	0.805 (0.63–0.979)	70% (34.8%–93.3%)	90.9% (58.7%–99.8%)	87.5% (47.3%–99.7%)	76.9% (46.2%–95%)
AG3 50–59 years	0.739 (0.602–0.876)	67.9% (47.6%–84.1%)	80% (51.9%–95.7%)	86.4% (65.1%–97.1%)	57.1% (34%–78.2%)
AG4 60–75 years	0.824 (0.0–1)	64.7% (38.3%–85.8%)	100% (2.5%–100%)	100% (71.5%–100%)	14.3% (0.361%–57.9%)
	Combined polyneuropathy sensory index (CPNSI) (4 out of 4; right and left sural <8.5, right and left superficial peroneal <4.5)				
Whole cohort	0.758 (0.688–0 .829)	54.4% (40.7%–67.6%)	97.3% (85.8%–99.9%)	96.9% (83.8%–99.9%)	58.1% (44.8%–70.5%)
AG1 <40 years	0.75 (0.26–1)	50% (1.26%–98.7%)	100% (69.2%–100%)	100% (2.5%–100%)	90.9% (58.7%–99.8%)
AG2 40–49 years	0.75 (0.587–0.913)	50% (18.7%–81.3%)	100% (71.5%–100%)	100% (47.8%–100%)	68.8% (41.3%–89%)
AG3 50–59 years	0.717 (0.602–0.831)	50% (30.6%–69.4%)	93.3% (68.1%–99.8%)	93.3% (68.1%–99.8%)	50% (30.6%–69.4%)
AG4 60–75 years	0.824 (0.0–1)	64.7% (38.3%–85.8%)	100% (2.5%–100%)	100% (71.5%–100%)	14.3% (0.361%–57.9%)

Abbreviation: CPNSI, combined polyneuropathy sensory index; NPV, negative predictive value; PPV, positive predictive value; ROC, receiver operator characteristic.

## 4 DISCUSSION

Our data show that the ranges of sensory action potentials among patients with normal scores on neuropathy‐assessment scales are consistent with previously reported normal ranges (Esper et al., 2005; Geney‐Castro et al., 2022; Lo et al., 2006; Overbeek et al., 2005). Our data have the advantage of using validated neuropathy‐screening clinical tools to determine the presence or absence of neuropathy and show the merit of using prospectively collected data. We described the sensitivity, specificity, PPV, and NPV, which were not described in many prior publications. Our data have the advantage of presenting and comparing the range of SNAPs in three separate groups and presenting diagnostic accuracy parameters in two different ways (including and excluding control participants). The inclusion of data from control participants adds to our understanding and produces a more complete picture about the extent of the subclinical neuropathy that could be present in participants with diabetes without clinical neuropathy, and it may affect the SNAP values and skew the results. In other words, if subclinical neuropathy was significant among individuals with diabetes without clinical neuropathy, we may not know if these diabetic neuropathies are normal or abnormal, unless we compare them to control participants. Our data showed that there is no difference between the SNAP values for the control participants and participants with diabetes without clinical neuropathy. Esper et al. (2005) found that the 5% LLN of SNA was 7 μV in 40–59‐year olds, which was similar to what we found among the control group (≥6 μV); in contrast, the 5% LLN value was >4 μV in our participants with diabetes without neuropathy. Furthermore, the 5% LLN of our participants aged ≥60 years was slightly higher than that reported by Esper et al. (7.5 vs. 3 μV). This could possibly be related to the use of standardized neuropathy scales in our study as a more robust method to differentiate between control participants and participants with clinical diabetic neuropathy. The sural nerve was measured in a cohort of 101 participants above the age of 60 years, with an average amplitude of 15 μV, which was higher than the average value observed in our control participants (7.5 μV), as well as in the participants with diabetes without neuropathy (6.6 μV) (Geney‐Castro et al., 2022). Lo et al. included 90 control participants and reported an average SPSA of 17.5 μV for participants aged 35–55 years and 6 μV for those above 55 years of age; this is higher than the median SPSA of 8 μV observed for the 40–59‐year AG in our study, and rather similar to the median value for our >60 years AG (5 μV). The difference in the younger AG could be attributed partially to the inclusion of younger participants (<40 years) in their control group, more so as they used the mean statistics to describe the average value, which is more sensitive to outliers/extreme values as compared to median statistics used in our study. Moreover, the lower limit of standard deviation they reported was 6.2 μV, which is comparable to our 25% LLN value of 6.6 and 5.7 μV for the 40–49 and 50–59 years AGs, respectively (Lo et al., 2006). The 5% LLN for the SRSA was reported to be 25 μV for the under‐40 AG, 17 μV for the 40–60 AG, and 12 μV for the over‐60 AG, which is consistent with our data, as shown in Table 2 (Esper et al., 2005). The 5% LLN of the SRAR was reported to be 0.2–0.21 regardless of age, which is consistent with the ratio of >= 0.2 obtained in all AGs in our cohort, despite the fact that the average was 0.36–0.43 in the under‐60‐year olds and over 0.2 in the over‐60‐year olds both in control participants and participants with diabetes without clinical neuropathy (Esper et al., 2005; Overbeek et al., 2005).

We found that a low SNA has a high specificity for neuropathy diagnosis, whether control participants were included (92.3%) or excluded (86%). The sensitivity of SNA was moderate (68%) and did not change with the exclusion of control participants. This may suggest that the effect of subclinical neuropathy on SNA is small. A low SPSA is sensitive for neuropathy (86%), regardless of whether control participants are included or excluded. The sensitivity of using a single nerve to diagnose neuropathy was reported in prior studies to be 75% for the sural nerve and 88% for the SPS nerve, which is similar to our results (Table 3) (Lo et al., 2006). Few studies have reported a 60%–66% sural nerve sensitivity for neuropathy (Kural et al., 2017; Rutkove et al., 1997). Most prior studies have focused on sensitivity, with limited data on specificity or predictive values. However, sural nerve specificity for detecting neuropathy was reported to be 85%–95%, with a PPV of 94%–98%, which is consistent with our data (Kural et al., 2017). The sensitivity of SRAR with a cutoff value of <0.4 was found to be 90%, which is much higher than that of our cohort (45%–50%); this may have occurred because we used a cutoff value of <0.2 to improve the specificity and PPV (Rutkove et al., 1997). Despite our data showing lower sensitivity, the NPV remains good at 72%. The reported specificity of SRAR (<0.21) was 96% (Sullivan et al., 2008), which is comparable to our results (100%).

We proposed a new simplified composite score, the CPNSI, to answer an important question—if the amplitude of 3–4 sensory nerves in lower limb is within the lower limits of normal (<25% LLN); can this be used to improve the diagnostic accuracy and the predictive values for diagnosing clinical neuropathy? We found that the CPNSI, which consists of four total points (one point for each of the following nerves if their amplitude is less than 25% of LLN, right sural, left sural, right SPS, and left SPS potentials), increases the PPV above 90% without compromising the NPV compared with using a single SNA (<90% PPV). We found that PPV improved the most in the below‐50 AG using the CPNSI compared to using a single SNA. A previous study found that using many NCS parameters improved sensitivity and specificity compared to using isolated NCS parameter (Dyck et al., 2003; Heise et al., 2012). Several studies have demonstrated that in addition to being more sensitive and reproducible, a composite score of nerve conduction has a higher correlation with polyneuropathy than a single‐nerve measurement (Dyck et al., 2003). Amplitude measurements were found to correlate more with neuropathy impairment than conduction velocity measurements. Combined scores are likely to minimize the random errors usually associated with single‐nerve parameters. The previously published composite scores shared the following challenges (Dyck et al., 2003; Heise et al., 2012). Their calculations were complex as both scores used a combination of conduction velocity, amplitude, and f‐waves for multiple nerves. Moreover, there was no simple cutoff value to calculate these scores that may make their use in clinical practice more difficult. Dyck et al. investigated several composite scores composed of different combinations of ulnar, peroneal, tibial, and SNAs and conduction velocities. Although it was sensitive (85%) for chronic inflammatory demyelinating polyneuropathy, it was poorly sensitive (30.9%) for diabetic polyneuropathy as compared to 90% sensitivity for a CPNSI score of 1% and 66.7% for a CPNSI score of 3 in our study (Dyck et al., 2003). This improvement in sensitivity values is probably due to the use of multiple nerves in the lower part of the normal range (<25% LLN) in the CPNSI. In another study that examined the composite scores of the sural nerve conduction velocity and amplitude, the superficial peroneal nerve amplitude, and the common peroneal nerve motor conduction velocity, a sensitivity of 74%–81% and a specificity of 97%–98% were reported (Heise et al., 2012). Although the reported values were higher than the values in our study, the patient selection in their groups was not based on a validated neuropathy scale, as they examined sensitivity among patients with suspected diabetic polyneuropathy, while using patients without diabetes referred to lumbosacral plexopathy to measure the specificity (Heise et al., 2012).

Our data provide some insight into the effect of aging on the normal SNAP amplitude and its diagnostic accuracy. We found that there is a decline with age in the sensory nerve amplitude among no clinical neuropathy participants with and without diabetes, including SNA, SPSA, SRSA, and SRAR (Table 2). This was recently studied in a large cohort of 3780 control participants (Taams et al., 2023). The researchers found a nonlinear decline in SNA in participants without neuropathy, with a more rapid decline after the age of 65 years. They found non‐recordable SNA in 1.8% and 25% in the 40–49 years and >80 years AGs, respectively. This effect was also reflected in the diagnostic accuracy in our data in the 60–65 years AG, as there was a drop in the NPV in this AG, although the PPV remained very high. The usual interpretation of such a finding is that SNAPs are excellent for confirming a diagnosis but not the best test to exclude a diagnosis in this AG. The increased prevalence of neuropathy among the older AGs could explain the high PPV and low NPV, as predictive values are dependent on disease prevalence. However, the other interpretation is that a low NPV among those aged >60 years could be attributed to overclassifying patients to the neuropathy group, using such current neuropathy scales as absent ankle reflex and reduced vibration sensation, which are commonly observed in participants without neuropathy, particularly after the age of 65 years (Taams et al., 2023). It was found that superficial pain sensation remained stable across this AG. This could support the argument that classifying participants as having neuropathy in the >65‐year AG should rely on methods that do not provide equal weight for absent ankle reflexes and vibration as to superficial pain sensation, and this may give a more accurate PPV and NPV in this AG.

Our study has several limitations. First, the effect of the height and weight of the participants was not measured; however, height explains only 6% of the amplitude variance (Rivner et al., 2001). On the other hand, we stratified the results according to age, which explains up to 15% of the amplitude variance and remains the main factor affecting amplitudes (Esper et al., 2005; Rivner et al., 2001). Second, although there is no gold standard test for diagnosing neuropathy, we used validated clinical scales, such as the UENS, for the diagnosis. Third, there were few participants (*n* = 4) above the age of 60 years classified in the no neuropathy group (UENS < 5) as compared to 17 participants with neuropathy (UENS ≥ 5), whereas, for the <40 years AG, there were more participants in the no neuropathy group (*n* = 10) as compared to the neuropathy group (*n* = 2). However, this reflects the fact that neuropathy is more prevalent among older AGs, and maintaining such prevalence in our data adds to its reliability, as diagnostic accuracy is influenced by the prevalence of the disease. Fourth, we did not include motor nerve conduction as a part of this study. Fifth, we did not include information about the use of neuropathic pain medications. Sixth, we determined painful neuropathy‐based solely on the participant's description of “burning feet,” and a subgroup analysis for painful feet was not performed. Despite these limitations, we believe that our data added more insights into the specificity and predictive values of sensory potentials in diagnosing neuropathy, which are often not described in the literature.

In conclusion, our data show that if three nerves were below the 25% lower limits of normal out of four nerves (bilateral sural and bilateral SPS potentials) that would provide high PPV s and specificities for neuropathy diagnoses. We described the sensitivity, specificity, negative, and PPV s for sensory nerves action potential in cohort classified into neuropathy and no neuropathy based on validated neuropathy scale measurements.

## AUTHOR CONTRIBUTIONS

*Study design; data acquisition and interpretation; statistical analysis and manuscript preparation*: Ahmad R. Abuzinadah. *Data acquisition and manuscript review*: Moafaq S. Alrawaili. *Study design and manuscript review*: Aysha A. Alshareef. *Data acquisition and manuscript review*: Hussien S. Alkully. *Study design; data acquisition and manuscript review*: Haneen Milyani. *Data acquisition and manuscript review*: Bashayr Alamri. *Study design; data acquisition and manuscript review*: Weam Alshora, Ahmed K. Bamaga.

## CONFLICT OF INTEREST STATEMENT

The authors declare that they have no known conflicts of interest or personal relationships that could have appeared to influence the work reported in this paper.

## FUNDING INFORMATION

This research did not receive any funding from public, commercial, or not‐for‐profit funding agencies.

### PEER REVIEW

The peer review history for this article is available at https://publons.com/publon/10.1002/brb3.3423.

## Supporting information

Supplementary Material

## Data Availability

All data are available upon direct request to corresponding author.
