# Supplementary material for: Values and diagnostic accuracy of sensory nerve action potentials in control participants and participants with diabetes with and without clinical diabetic neuropathy, based on neuropathy scale measurements
Source: Brain Behav. 2024 Feb 13;14(2):e3423. doi: 10.1002/brb3.3423 (PMC10864687; doi:10.1002/brb3.3423)
Supplement: Supplementary file 1 — Supplementary Material [file BRB3-14-e3423-s001.docx]

# **Supplementary materials: appendix A.**

We also re-performed all the analysis, including the six participants who had UENS <5 but whose MNSI was >7.5. These participants were included in the diabetic without neuropathy group.

# **eTable 1. Participants’ characteristics**

# **eTable 2. Range of sensory potentials parameters in control participants and diabetic participants with and without clinical diabetic neuropathy**

# **eTable 3. Diagnostic accuracy of the amplitude of the sensory nerve action potentials. (No neuropathy group included control participants and participants with diabetes without neuropathy)**

# **eTable 4. Diagnostic accuracy of the amplitude of the sensory nerve action potentials. (No neuropathy included only participants with diabetes without neuropathy and excluded controls participants)**

# **eTable 1. Participants’ characteristics**

|  | **No neuropathy (G1)** (control participants) N=41 | **No neuropathy (G2)**(participants with diabetes)N=43 | **Diabetic Neuropathy (G3)** N=57 | **G1 vs G3*** | **G2 vs G3*** | **All groups**** |
| --- | --- | --- | --- | --- | --- | --- |
| Age, median (IQR) | 51 (45-56) | 46 (40-56) | 54 (51-61) | 0.0103 | 0.0004 | 0.0008 |
| Male, n(%) | 19 (46.3) | 19 (44.19) | 32 (56.14) | 0.226 | 0.163 | 0.45 |
| Diabetes, n(%) | 0 | 43 (100) | 57 (100) | -- | ---- | 0.000 |
| Diabetes duration (years), median (IQR) | 0 | 5.5 (2-12) | 15 (10-21.5) | -- | 0.000 | 0.0001 |
| UENS, median (IQR) | 0 (0-0) | 0 (0-2) | 10 (6-16) | 0.000 | 0.000 | 0.0001 |
| MNSI, median (IQR) | 0 (0-1.5) | 3 (1.5-6) | 10.5 (8-13) | 0.000 | 0.000 | 0.0001 |
| Age group1: 60-75 n(%) | 3 (7.3) | 2 (4.65) | 17 (29.8) | 0.003 | 0.000 | 0.000 |
| Age group2: 50-59 n(%) | 20 (48.8) | 15 (34.88) | 28 (49.12) |  |  |  |
| Age group3: 40-49 n(%) | 18 (43.9) | 16 (37.21) | 10 (17.5) |  |  |  |
| Age group4: <40 n(%) | 0 | 10 (23.26) | 2 (3.5) |  |  |  |

* Mann-Whitney test, Fisher's exact test for proportion comparisons

**Kruskal-Wallis rank test for median comparisons, Fisher's exact test for proportion comparisons

G1 vs G2 comparison p value were >0.05 except UENS, MNSI comparison and age group p values were 0.046, 0.00, and 0.005, respectively.

# **eTable 2 Range of sensory potentials parameters in control participants and diabetic participants with and without clinical diabetic neuropathy**

| Age group | No neuropathy (G1) (control participants),  µV median (IQR)/5% LLN | No neuropathy (G2) (participants with diabetes),  µV median (IQR)/5% LLN | Diabetic Neuropathy (G3), µV median (IQR) | P value^*^  G1 vs G3 | P value^8^  G2 vs G3 | P value** (all groups) |
| --- | --- | --- | --- | --- | --- | --- |
|  | Sural nerve potential (µV) | | | | | |
| Whole cohort | 11.9 (9.15-15.25)/7.4 | 10.5 (8.5-15.4)/3.95 | 4.05 (0-8.95) | 0.000 | 0.000 | 0.0001 |
| Group1 < 40 years | --- | 15.25 (10.1-21.8)/5.95 | 8.18 (0-16.35) | ---- | 0.39 | 0.39 |
| Group2 40-49 years | 13.38 (9.95-17.35)/7.55 | 10.45 (7.78-13.15)/0 | 2.03 (0-7.9) | 0.0025 | 0.024 | 0.0029 |
| Group3 50-59 years | 12.2 (8.68-14.63)/6 | 8.5 (8.5-14.2)/3.95 | 4.37 (0-9.58) | 0.0001 | 0.004 | 0.0002 |
| Group4 60-75 years | 7.55 (7.5-11.6)/7.5 | 9.87 (6.65-13.1)/6.65 | 0 (0-8.75) | 0.201 | 0.206 | 0.249 |
|  | Superficial peroneal sensory nerve potential (µV) | | | | | |
| Whole cohort | 7.95 (6.25-10)/3.65 | 5.5 (3.55-10.5)/0.19 | 0 (0-4.3) | 0.000 | 0.000 | 0.0001 |
| Group1 < 40 years | --- | 8.7 (4.65-10.6)/2.5 | 1.88 (0-3.75) | --- | 0.0857 | 0.0857 |
| Group2 40-49 years | 8.4 (6.65-10)/3.65 | 5.8 (2.75-9.03)/0 | 3.18 (0-4) | 0.0005 | 0.0497 | 0.0015 |
| Group3 50-59 years | 7.7 (5.7-9.95)/3.85 | 5.05 (3.55-13.5)/1.4 | 1.5(0-4.33) | 0.000 | 0.0015 | 0.0001 |
| Group4 60-75 years | 5.07 (0.83-9.3)/0.83 | 4.9 (4.65-5.15)/4.65 | 0(0-5.3) | 0.125 | 0.283 | 0.232 |
|  | Superficial radial sensory nerve potential (µV) | | | | | |
| Whole cohort | 29.8 (26.3-37.55)/20.7 | 28.48 (23.1-40.3)/18.05 | 18.1 (10.85-26.55) | 0.000 | 0.000 | 0.0001 |
| Group1 < 40 years | ---- | 32.3 (26.9-42.8)/21.8 | 16.4 (6.5-26.2) | --- | 0.099 | 0.099 |
| Group2 40-49 years | 30.4 (26.3-40.5)/18.8 | 28.1 (24.3-37.1)/20 | 19.3 (10.2-33.2) | 0.0214 | 0.0452 | 0.0466 |
| Group3 50-59 years | 28.6 (25.9-32.1)/21.7 | 25.7 (18.1-40.9)/14.7 | 18.7 (11.3-27.6) | 0.0022 | 0.0128 | 0.0027 |
| Group4 60-75 years | 37.6 (37.6-37.6)/ 37.6 | 29.3 (24.9-33.8)/24.9 | 13.5 (10.9-20.9) | 0.1069 | 0.0617 | 0.0571 |
|  | Sural to radial ratio | | | | | |
| Whole cohort | 0.39 (0.33-0.51)/0.22 | 0.36 (0.27-0.45)/0.21 | 0.21 (0-0.41) | 0.0002 | 0.0014 | 0.0001 |
| Group1 < 40 years | --- | 0.36 (0.31-0.74)/ 0.27 | 0.31 (0-0.62) | --- | 0.4795 | 0.4795 |
| Group2 40-49 years | 0.42 (0.35-0.54)/0.22 | 0.36 (0.25-0.43)/0 | 0.11 (0-0.32) | 0.0011 | 0.0242 | 0.0019 |
| Group3 50-59 years | 0.4 (0.29-0.51)/0.27 | 0.4 (0.31-0.46)/0.21 | 0.22 (0-0.46) | 0.0209 | 0.0534 | 0.0327 |
| Group4 60-75 years | 0.2 (0.2-0.2)/0.2 | 0.32 (0.26-0.38)/0.26 | 0.15 (0-0.41) | 1.0 | 0.7039 | 0.8559 |

IQR : interquartile range. LLN: lower limit of normal.

^*^Wilcoxon rank-sum (Mann-Whitney) test

^**^ Kruskal-Wallis test

All comparison between G1 and G2 were statistically insignificant at >0.05.

# **eTable 3. Diagnostic accuracy of the amplitude of the sensory nerve action potentials. (No neuropathy group included control participants and participants with diabetes without neuropathy)**

| Age group | ROC | Sensitivity | Specificity | PPV | NPV |
| --- | --- | --- | --- | --- | --- |
|  | Single sural nerve potential <5.1 µV | | | | |
| Whole Cohort | 0.794  (0.726- 0.863) | 68.4%  (54.8%- 80.1%) | 90.5%  (82.1%- 95.8%) | 83%  (69.2%- 92.4%) | 80.9%  (71.4%- 88.2%) |
| Age group1 < 40 Years | 0.7  (0.2 1) | 50%  (1.26%- 98.7%) | 90%  (55.5%- 99.7%) | 50%  (1.26%- 98.7%) | 90%  (55.5%- 99.7%) |
| Age group2 40-49 Years | 0.841  (0.699- 0.983) | 80%  (44.4%- 97.5%) | 88.2%  (72.5%- 96.7%) | 66.7%  (34.9%- 90.1%) | 93.8%  (79.2%- 99.2%) |
| Age group3 50-59 Years | 0.796  (0.697- 0.896) | 67.9%  (47.6%- 84.1%) | 91.4%  (76.9%- 98.2%) | 86.4%  (65.1%- 97.1%) | 78%  (62.4% -89.4%) |
| Age group4 60-75 Years | 0.824  (0.706- 0.941) | 64.7%  (38.3% -85.8%) | 100%  (47.8%- 100%) | 100%  (71.5%- 100%) | 45.5%  (16.7%- 76.6%) |
|  | Single superficial sensory peroneal < 4.5 µV | | | | |
| Whole Cohort | 0.712  (0.64- 0.784) | 86%  (74.2%- 93.7%) | 56.4%  (44.7%- 67.6%) | 59%  (47.7%- 69.7%) | 84.6%  (71.9%- 93.1%) |
| Age group1 < 40 Years | 0.8  (0.64-0.96) | 100%  (15.8%- 100%) | 60%  (26.2%- 87.8%) | 33.3%  (4.33%- 77.7%) | 100%  (54.1%- 100%) |
| Age group2 40-49 Years | 0.759  (0.63- 0.887) | 90%  (55.5%-99.7%) | 61.8%  (43.6%- 77.8%) | 40.9%  (20.7%- 63.6%) | 95.5%  (77.2%- 99.9%) |
| Age group3 50-59 Years | 0.679  (0.566- 0.791) | 85.7%  (67.3%- 96%) | 50%  (31.3%- 68.7%) | 61.5%  (44.6%- 76.6%) | 78.9%  (54.4%- 93.9%) |
| Age group4 60-75 Years | 0.662  (0.364- 0.96) | 82.4%  (56.6%- 96.2%) | 50%  (6.76%- 93.2%) | 87.5%  (61.7%- 98.4%) | 40%  (5.27%- 85.3%) |
|  | Sural to radial ratio <0.2 | | | | |
| Whole Cohort | 0.727  (0.654- 0.799) | 48%  (33.7%- 62.6%) | 97.3%  (90.7%- 99.7%) | 92.3%  (74.9%- 99.1%) | 73.7%  (63.9%- 82.1%) |
| Age group1 < 40 Years | 0.75  (0.26- 1) | 50%  (1.26%- 98.7%) | 100%  (66.4%- 100%) | 100%  (2.5%- 100%) | 90%  (55.5%- 99.7%) |
| Age group2 40-49 Years | 0.721  (0.552- 0.889) | 50%  (18.7%- 81.3%) | 94.1%  (80.3%- 99.3%) | 71.4%  (29% -96.3%) | 86.5%  (71.2%- 95.5%) |
| Age group3 50-59 Years | 0.731  (0.633- 0.828) | 46.2%  (26.6%- 66.6%) | 100%  (88.1%- 100%) | 100%  (73.5%- 100%) | 67.4%  (51.5%- 80.9%) |
| Age group4 60-75 Years | 0.75  (0.602- 0.898) | 50%  (21.1%- 78.9%) | 100%  (29.2%- 100%) | 100%  (54.1%- 100%) | 33.3%  (7.49%- 70.1%) |
|  | combined polyneuropathy sensory index (CPNSI) (right and left sural <8.5 µV, right and left superficial peroneal <4.5 µV) | | | | |
| 1 Out Of 4 Sensory Nerves | 0.655  (0.589- 0.721) | 91.2%  (80.7%- 97.1%) | 39.7%  (28.8%- 51.5%) | 52.5%  (42.2%- 62.7%) | 86.1%  (70.5%- 95.3%) |
| 2 Out Of 4 Nerves | 0.756  (0.684- 0.829) | 80.7%  (68.1%- 90%) | 70.5%  (59.1%- 80.3%) | 66.7%  (54.3%- 77.6%) | 83.3%  (72.1%- 91.4%) |
| 3 Out Of 4 Sensory Nerves | 0.788  (0.719- 0.858) | 66.7%  (52.9%- 78.6%) | 91%  (82.4%- 96.3%) | 84.4%  (70.5%- 93.5%) | 78.9%  (69%- 86.8%) |
| 4 Out Of 4 Nerves | 0.753  (0.684- 0.821) | 54.4%  (40.7% -67.6%) | 96.2%  (89.2%- 99.2%) | 91.2%  (76.3%- 98.1%) | 74.3%  (64.6%- 82.4%) |
|  | combined polyneuropathy sensory index (CPNSI) (3 out of 4; right and left sural <8.5 µV, right and left superficial peroneal <4.5 µV) | | | | |
| Whole Cohort | 0.788  (0.719- 0.858) | 66.7%  (52.9%- 78.6%) | 91%  (82.4%- 96.3%) | 84.4%  (70.5%- 93.5%) | 78.9%  (69% -86.8%) |
| Age group1 < 40 Years | 0.75  (0.26- 1) | 50%  (1.26%- 98.7%) | 100%  (69.2%- 100%) | 100%  (2.5%- 100%) | 90.9%  (58.7%- 99.8%) |
| Age group2 40-49 Years | 0.791  (0.632- 0.951) | 70%  (34.8%- 93.3%) | 88.2%  (72.5%- 96.7%) | 63.6%  (30.8%- 89.1%) | 90.9%  (75.7%- 98.1%) |
| Age group3 50-59 Years | 0.789  (0.686- 0.893) | 67.9%  (47.6%- 84.1%) | 90%  (73.5%- 97.9%) | 86.4%  (65.1%- 97.1%) | 75%  (57.8%- 87.9%) |
| Age group4 60-75 Years | 0.824  (0.706- 0.941) | 64.7%  (38.3%- 85.8%) | 100%  (39.8%- 100%) | 100%  (71.5%- 100%) | 40%  (12.2%- 73.8%) |
|  | combined polyneuropathy sensory index (CPNSI) (4 out of 4; right and left sural <8.5 µV, right and left superficial peroneal <4.5 µV) | | | | |
| Whole Cohort | 0.753  (0.684- 0.821) | 54.4%  (40.7%- 67.6%) | 96.2%  (89.2%- 99.2%) | 91.2%  (76.3%- 98.1%) | 74.3%  (64.6%- 82.4%) |
| Age group1 < 40 Years | 0.75  (0.26- 1) | 50%  (1.26%- 98.7%) | 100%  (69.2%- 100%) | 100%  (2.5%- 100%) | 90.9%  (58.7%- 99.8%) |
| Age group2 40-49 Years | 0.721  (0.552- 0.889) | 50%  (18.7%- 81.3%) | 94.1%  (80.3%- 99.3%) | 71.4%  (29% -96.3%) | 86.5%  (71.2%- 95.5%) |
| Age group3 50-59 Years | 0.733  (0.634- 0.833) | 50%  (30.6%- 69.4%) | 96.7%  (82.8%- 99.9%) | 93.3%  (68.1%- 99.8%) | 67.4%  (51.5%- 80.9%) |
| Age group4 60-75 Years | 0.824  (0.706- 0.941) | 64.7%  (38.3%- 85.8%) | 100%  (39.8%- 100%) | 100%  (71.5%- 100%) | 40%  (12.2%- 73.8%) |

# **eTable 4. Diagnostic accuracy of the amplitude of the sensory nerve action potentials. (No neuropathy included only participants with diabetes without neuropathy and excluded controls participants)**

| Age group | ROC | Sensitivity | Specificity | PPV | NPV |
| --- | --- | --- | --- | --- | --- |
|  | Single sural nerve potential <5.1 µV | | | | |
| Whole cohort | 0.761  (0.678-0.843) | 68.4%  (54.8%- 80.1%) | 83.7%  (69.3%- 93.2%) | 84.8%  (71.1%- 93.7%) | 66.7%  (52.5%-78.9%) |
| Age group1 < 40 years | 0.7  (0.2-1) | 50%  (1.26% 98.7%) | 90%  (55.5%- 99.7%) | 50%  (1.26%- 98.7%) | 90%  (55.5%- 99.7%) |
| Age group2 40-49 years | 0.775  (0.604- 0.946) | 80%  (44.4%-97.5%) | 75%  (47.6%- 92.7%) | 66.7%  (34.9%- 90.1%) | 85.7%  (57.2%-98.2%) |
| Age group3 50-59 years | 0.773  (0.647-0 .898) | 67.9%  (47.6%- 84.1%) | 86.7%  (59.5%- 98.3%) | 90.5%  (69.6%- 98.8%) | 59.1%  (36.4%- 79.3%) |
| Age group4 60-75 years | 0.824  (0.706- 0.941) | 64.7%  (38.3%- 85.8%) | 100%  (15.8%- 100%) | 100%  (71.5%- 100%) | 25%  (3.19%- 65.1%) |
|  | Single superficial sensory peroneal < 4.5 µV | | | | |
| Whole cohort | 0.662  (0.574- 0.75) | 86%  (74.2%- 93.7%) | 46.5%  (31.2%- 62.3%) | 68.1%  (56%- 78.6%) | 71.4%  (51.3%- 86.8%) |
| Age group1 < 40 years | 0.8  (0.64-0.96) | 100%  (15.8%- 100%) | 60%  (26.2%- 87.8%) | 33.3%  (4.33%-77.7%) | 100%  (54.1%- 100%) |
| Age group2 40-49 years | 0.669  (0 .51- 0.828) | 90%  (55.5%- 99.7%) | 43.8%  (19.8%-70.1%) | 50%  (26%- 74%) | 87.5%  (47.3%- 99.7%) |
| Age group3 50-59 years | 0.629  (0.484- 0.773) | 85.7%  (67.3%- 96%) | 40%  (16.3%-67.7%) | 72.7%  (54.5%-86.7%) | 60%  (26.2%-87.8%) |
| Age group4 60-75 years | 0.662  (0.163-1) | 82.4%  (56.6%- 96.2%) | 50%  (1.26%-98.7%) | 93.3%  (68.1%- 99.8%) | 25%  (0.631%- 80.6%) |
|  | Sural to radial ratio <0.2 | | | | |
| Whole cohort | 0.716  (0.639-0.793) | 48%  (33.7%- 62.6%) | 95.2%  (83.8%-99.4%) | 92.3%  (74.9%-99.1%) | 60.6%  (47.8%- 72.4%) |
| Age group1 < 40 years | 0.75  (0.26-1) | 50%  (1.26%- 98.7%) | 100%  (66.4%- 100%) | 100%  (2.5%- 100%) | 90%  (55.5%- 99.7%) |
| Age group2 40-49 years | 0.688  (0.504-0.871) | 50%  (18.7%- 81.3%) | 87.5%  (61.7%- 98.4%) | 71.4%  (29%-96.3%) | 73.7%  (48.8%-90.9%) |
| Age group3 50-59 years | 0.731  (0.633-0.828) | 46.2%  (26.6%-66.6%) | 100%  (78.2%- 100%) | 100%  (73.5%- 100%) | 51.7%  (32.5%- 70.6%) |
| Age group4 60-75 years | 0.75  (0.602-0.898) | 50%  (21.1%- 78.9%) | 100%  (15.8%- 100%) | 100%  (54.1%-100%) | 25%  (3.19%-65.1%) |
|  | combined polyneuropathy sensory index (CPNSI) (right and left sural <8.5 µV, right and left superficial peroneal <4.5 µV) | | | | |
| 1 out of 4 sensory nerves | 0.619  (0.539-0.699) | 91.2%  (80.7%- 97.1%) | 32.6%  (19.1%- 48.5%) | 64.2%  (52.8%-74.6%) | 73.7%  (48.8%- 90.9%) |
| 2 out of 4 nerves | 0.717  (0.628-0.807) | 80.7%  (68.1%- 90%) | 62.8%  (46.7%-77%) | 74.2%  (61.5%- 84.5%) | 71.1%  (54.1%- 84.6%) |
| 3 out of 4 sensory nerves | 0.752  (0.669- 0.835) | 66.7%  (52.9%- 78.6%) | 83.7%  (69.3%- 93.2%) | 84.4%  (70.5%- 93.5%) | 65.5%  (51.4%- 77.8%) |
| 4 out of 4 nerves | 0.737  (0.661- 0.813) | 54.4%  (40.7%- 67.6%) | 93%  (80.9%- 98.5%) | 91.2%  (76.3%- 98.1%) | 60.6%  (47.8%- 72.4%) |
|  | combined polyneuropathy sensory index (CPNSI) (3 out of 4; right and left sural <8.5 µV, right and left superficial peroneal <4.5 µV) | | | | |
| Whole cohort | 0.752  (0.669- 0.835) | 66.7%  (52.9%- 78.6%) | 83.7%  (69.3%- 93.2%) | 84.4%  (70.5%- 93.5%) | 65.5%  (51.4%- 77.8%) |
| Age group1 < 40 years | 0.75  (0.26 -1) | 50%  (1.26%- 98.7%) | 100%  (69.2%-100%) | 100%  (2.5%- 100%) | 90.9%  (58.7%- 99.8%) |
| Age group2 40-49 years | 0.725  (0.539- 0.911) | 70%  (34.8%- 93.3%) | 75%  (47.6%- 92.7%) | 63.6%  (30.8%- 89.1%) | 80%  (51.9%- 95.7%) |
| Age group3 50-59 years | 0.739  (0.602-0.876) | 67.9%  (47.6%- 84.1%) | 80%  (51.9%- 95.7%) | 86.4%  (65.1%- 97.1%) | 57.1%  (34%- 78.2%) |
| Age group4 60-75 years | 0.824  (0.706- 0.941) | 64.7%  (38.3%-85.8%) | 100%  (15.8%-100%) | 100%  (71.5%- 100%) | 25%  (3.19%-65.1%) |
|  | combined polyneuropathy sensory index (CPNSI) (4 out of 4; right and left sural <8.5 µV, right and left superficial peroneal <4.5 µV) | | | | |
| Whole cohort | 0.737  (0.661- 0.813) | 54.4%  (40.7%- 67.6%) | 93%  (80.9%- 98.5%) | 91.2%  (76.3%- 98.1%) | 60.6%  (47.8%- 72.4%) |
| Age group1 < 40 years | 0.75  (0.26- 1) | 50%  (1.26%- 98.7%) | 100%  (69.2%- 100%) | 100%  (2.5%-100%) | 90.9%  (58.7%- 99.8%) |
| Age group2 40-49 years | 0.688  (0.504- 0.871) | 50%  (18.7%- 81.3%) | 87.5%  (61.7%- 98.4%) | 71.4%  (29%- 96.3%) | 73.7%  (48.8%- 90.9%) |
| Age group3 50-59 years | 0.717  (0.602- 0.831) | 50%  (30.6%- 69.4%) | 93.3%  (68.1%- 99.8%) | 93.3%  (68.1%- 99.8%) | 50%  (30.6%- 69.4%) |
| Age group4 60-75 years | 0.824  (0.706- 0.941) | 64.7%  (38.3%- 85.8%) | 100%  (15.8%- 100%) | 100%  (71.5%- 100%) | 25%  (3.19%- 65.1%) |
